# Gilbert damping in CoFeB/GaAs(001) film with enhanced in-plane uniaxial magnetic anisotropy

**DOI:** 10.1038/srep43971

**Published:** 2017-03-06

**Authors:** H. Q. Tu, B. Liu, D. W. Huang, X. Z. Ruan, B. You, Z. C. Huang, Y. Zhai, Y. Gao, J. Wang, L. J. Wei, Y. Yuan, Y. B. Xu, J. Du

**Affiliations:** 1 National Laboratory of Solid State Microstructures and Department of Physics, Nanjing University, Nanjing 210093, P. R. China; 2 Department of Mathematics and Physics, Nanjing Institute of Technology, Nanjing 211167, P. R. China; 3 School of Electronic Science and Engineering, Nanjing University, Nanjing 210046, P. R. China; 4 Collaborative Innovation Center of Advanced Microstructures, Nanjing 210093, P. R. China; 5 Department of Physics and Jiangsu Key Laboratory of Advanced Metallic Materials, Southeast University, Nanjing 211189, P. R. China

## Abstract

A 3.5 nm amorphous CoFeB film was sputtered on GaAs (001) wafer substrate without applying magnetic field during deposition, and a significant in-plane uniaxial magnetic anisotropy (UMA) field (*H*_u_) of about 300 Oe could be achieved. To precisely determine the intrinsic Gilbert damping constant (*α*) of this film, both ferromagnetic resonance (FMR) and time-resolved magneto-optical Kerr effect (TRMOKE) techniques were utilized. With good fitting of the dynamic spectra of FMR and TRMOKE, *α* is calculated to be 0.010 and 0.013, respectively. Obviously, the latter is 30% larger than the former, which is due to the transient heating effect during the TRMOKE measurement. In comparison with ordinary amorphous CoFeB films with negligible magnetic anisotropies, *α* is enhanced significantly in the CoFeB/GaAs(001) film, which may be mainly resulted from the enhanced spin-orbit coupling induced by the CoFeB/GaAs interface. However, the significant in-plane UMA plays minor role in the enhancement of *α*.

In recent years, amorphous CoFeB thin film has been extensively studied owing to its promising application for spintronic devices^1^,^2^,^3^,^4^,^5^,^6^,^7^,^8^,^9^,^10^,^11^,^12^,^13^,^14^,^15^,^16^,^17^,^18^. Firstly, the amorphous CoFeB film deposited on appropriate metal or insulator film can produce prominent perpendicular magnetic anisotropy (PMA) (e.g. CoFeB/Ta, CoFeB/MgO) and may become perpendicularly magnetized under some certain circumstances^1^. Secondly, extremely large tunneling magnetoresistance (TMR) ratio and the spin transfer torque (STT) phenomenon in CoFeB-MgO-CoFeB magnetic tunnel junctions (MTJs) can be obtained^2^. Thirdly, for the purpose of generation and/or detection of spin current in the very recent studies, CoFeB has also been used to investigate the spin pumping effect^3^,^4^,^5^,^6^, (inverse) spin Hall effect^6^,^7^,^8^, spin Seebeck effect^8^,^9^, Nernst effect^9^ and so on. Finally, because the amorphous ferromagnetic (FM) film is expected to decrease the amount of pinning centers which are harmful for domain wall motion, the utilization of CoFeB may greatly increase the magnetization switching speed, which has potential application for high-performance magnetic random access memory (MRAM) devices^10^. Since the Gilbert damping is much correlated to the magnetization switching, it needs to be studied elaborately in amorphous CoFeB thin films.

To our knowledge, determination of the intrinsic Gilbert damping constant (*α*) of amorphous CoFeB film in various stacking structures has already been carried out. On the one hand, for the CoFeB films with weak in-plane uniaxial magnetic anisotropy (UMA), usually *α* was relatively small varying from 0.004 to 0.008 when the CoFeB films were deposited on different substrates with various thicknesses^11^,^12^,^13^,^14^. Only in a special case, it was reported that *α* could reach 0.013 in the Co_40_Fe_40_B_20_ films with in-plane anisotropic field (*H*_k_) to be 50 Oe after the CoFeB films were deposited on Si(100) substrates in magnetic field^15^. In these above studies, *α* was usually determined by ferromagnetic resonance (FMR) technique. On the other hand, for ultrathin (1.0~1.3 nm) CoFeB film in the MgO-based MTJs with significant PMA, *α* was usually reported in the range from 0.013 to 0.027, which was determined by FMR or time-resolved magneto-optical Kerr effect^1^,^16^,^17^,^18^ (TRMOKE) technique. And the values of *α* are generally larger than those reported in the cases of in-plane UMA. These above studies clearly show that *α* depends strongly on the CoFeB film thickness^13^ while weakly on the Co or Fe concentration with the composition of B fixed at 20%^16^. Although FMR and TRMOKE can be both employed to determine the value of *α*, since the magnetization precession is excited by different ways, i.e. microwave for FMR and femtosecond laser for TRMOKE, it is not clear whether or not the value of *α* determined by these two ways are the same. Therefore, the large diversity of *α* may be resulted from the thicknesses, magnetic anisotropies, capping or underneath layers for the CoFeB films, and the measurement methods as well.

Very recently, a significant and pure in-plane UMA could be achieved in amorphous CoFeB films when they were deposited on semiconductor (e.g. GaAs) films^19^,^20^. Note that this kind of UMA is not accompanied with any other multifold anisotropies, such as four-fold magneto-crystalline anisotropy in ultrathin Fe film^21^. In our latest work^22^, an enhanced pure in-plane UMA with the largest anisotropic field (*H*_u_~300 Oe) up to date could be obtained in the CoFeB film when it was deposited *directly* onto the GaAs(001) wafer substrate after proper pretreatments. Here, CoFeB refers to Co_56_Fe_24_B_20_. It needs to be emphasized that no magnetic field was applied during deposition of the CoFeB film. Although the inherent mechanism responsible for this kind of special UMA remains unclear yet, it is tentatively attributed to interfacial anelastic strains based on the bond-orientational anisotropy model^20^,^23^. However, to our knowledge, no effort has been made to investigate the Gilbert damping on these CoFeB films. In order to obtain more reliable and precise value of *α*, both FMR and TRMOKE techniques were employed simultaneously on this kind of CoFeB films.

## Results

**Figure 1:**
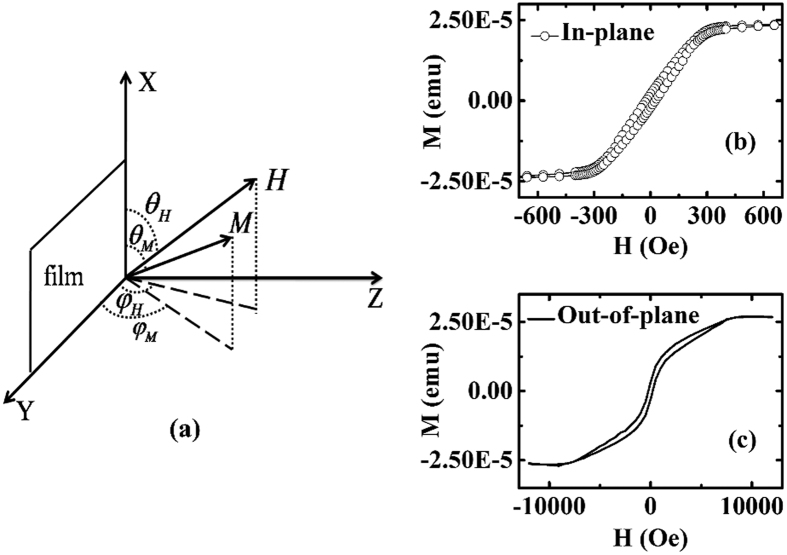
The coordinate system illustrating the FMR and TRMOKE measurement configurations (**a**), the in-plane (**b**) and out-of-plane (**c**) *M*-*H* loops for the CoFeB/GaAs(001) film.

The magnetization dynamics are generally described by the Landau-Lifshitz-Gilbert (LLG) equation as


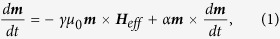


where ***m***is the unit magnetization vector, ***H***_eff_ is the effective magnetic field. Figure 1(a) shows the coordinate system to describe the FMR and TRMOKE measurement configurations. In the case of enhanced in-plane UMA, the total energy per unit volume can be written as


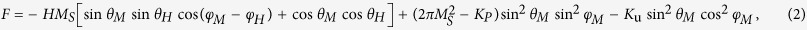


where the densities of Zeeman energy, effective demagnetized energy, and in-plane uniaxial anisotropy energy are described in the first, second and third terms, respectively; *M*_s_, *K*_P_ and *K*_u_ denote the saturate magnetization, out-of-plane and in-plane uniaxial anisotropic energy constants, respectively. According to Eq. (1) and Eq. (2), the precession frequency *f*, the reversal lifetime *τ* and the equilibrium equation can be derived to be^17^,^18^


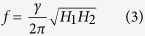



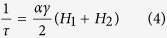



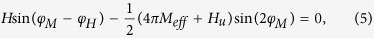


where 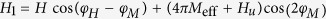, 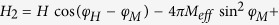 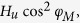

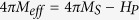
. The gyromagnetic ratio is 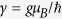. Here, *g* and *α* are Lande’s factor and intrinsic Gilbert damping constant, respectively.

As we all know, although both FMR and TRMOKE can determine the intrinsic Gilbert damping constant by proper fitting calculations, the result may be deviated from the true value due to too many parameters used in the fitting procedures. Therefore, in order to verify the validity of the fitted results, the static magnetic property of the CoFeB film needs to be characterized firstly for providing some reference parameters, such as *M*_s_, *H*_u_ and 4π*M*_eff_. The *M*-*H* loops for the in-plane and out-of-plane configurations are shown in Fig. 1(b) and (c), respectively. According to these *M*-*H* loops and the CoFeB film thickness, *M*_s_ is calculated to be about 1150 emu/cm^3^, and the saturation fields for in-plane and out-of-plane cases are about 300 Oe and 8500 Oe, respectively. Considering that the in-plane magnetizing process conforms to coherent rotation model approximately, *H*_u_ is equal to the in-plane saturation field^20^, i.e. *H*_u_~300 Oe. Finally, 4π*M*_eff_ is equal to the out-of-plane saturation field, i.e. 4π*M*_eff_~8500 Oe, and therefore *H*_P_~5944 Oe.

**Figure 2:**
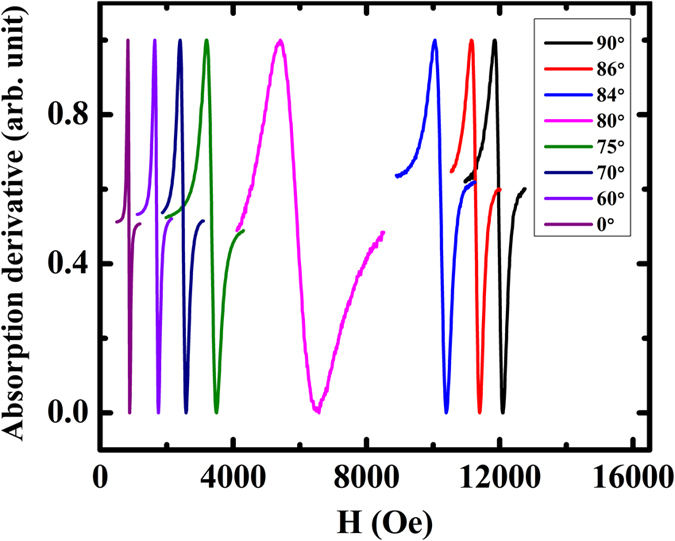
The out-of-plane FMR spectra in the form of derivative absorption for the CoFeB/GaAs(001) film.

The out-of-plane FMR spectra in the form of derivative absorption are recorded at 9.78 GHz, as shown in Fig. 2. Because this kind of FMR spectrum usually does not have a standard dispersive line shape, the resonance field (*H*_r_) and full width at half maximum (FWHM) (Δ*H*) can be obtained accurately by fitting the derivative absorption FMR spectrum with both the symmetric and asymmetric parts L (Lorenz) and D (Dispersive) as described by the following equation^24^


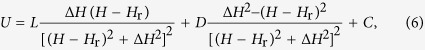


while the relationship between Δ*H*_*pp*_ and Δ*H* can be expressed as


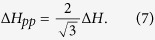


**Figure 3:**
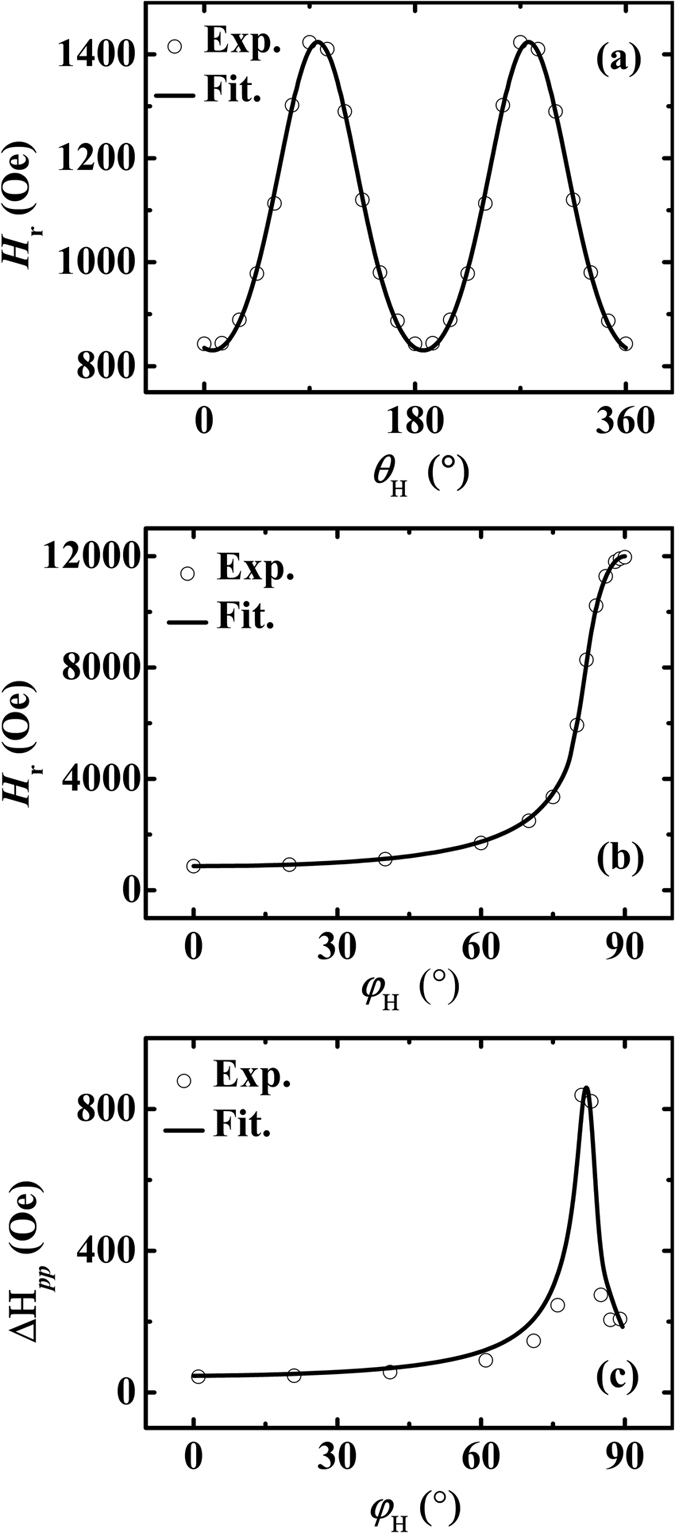
(**a**) The in-plane angular dependent resonance field, (**b**) the out-of-plane angular dependent resonance field and (**c**) the angular dependent linewidth of the out-of-plane FMR spectra for the CoFeB/GaAs(001) film. The open dots and solid lines represent the experimental and the fitted results, respectively.

Here, Δ*H*_*pp*_ refers to the peak-to-peak field variation in the FMR spectrum with standard asymmetric line shape. The angular dependent FMR spectra were recorded by varying the angle between the applied field and the in-plane UMA easy axis which is along the [110] direction. Both the experimental and fitted angular dependent resonance fields for in-plane and out-of-plane geometries are displayed in Fig. 3(a,b), respectively. The fitted curves are obtained by using the least square method based on the Eqs (3, 4, 5) mentioned above. The perfect fitting in Fig. 3(a) indicates a well-defined UMA in the film plane. All the fitted parameters are listed in Table 1. Note that no matter the fitting is performed on the in-plane or out-of-plane geometry, the two values of *H*_u_ (or 4π*M*_eff_, *g*) are closed to each other. Moreover, the fitted values for *H*_u_ and 4π*M*_eff_ are also approximately equal to those obtained from the *M*-*H* loop measurements. All these results indicate that the fitted parameters of *H*_u_, 4π*M*_eff_ and *g* listed in Table 1 are trustable.

Besides *H*_r_, Δ*H*_*pp*_ is another important ingredient of the FMR spectrum, which is much correlated the magnetic damping. As shown in Fig. 3(c), the experimental data of out-of-plane angular dependent Δ*H*_*pp*_ of the CoFeB film are indicated by the dots. It can be seen that Δ*H*_*pp*_ increases with increasing *φ*_H_ slowly when *φ*_H_ < 60°. After that, Δ*H*_*pp*_ increases abruptly and reaches a peak at about *φ*_H_ = 82°. Finally, Δ*H*_*pp*_ decreases quickly with further increasing *φ*_H_ up to 90°. The significant increase of Δ*H*_*pp*_ at intermediate angle is due to magnetic dragging effect^13^. Besides the intrinsic contribution which comes from the spin-orbit coupling, in the present study, the extrinsic contributions to Δ*H*_*pp*_ are considered as the fluctuation of 4π*M*_eff_ and *φ*_H_ which is caused by the magnetic inhomogeneities in the film, and the significant in-plane UMA as well. By inputting the parameters of 4π*M*_eff_, *g* and *H*_u_ fitted from the out-of-plane angular dependence of *H*_r_, the experimental out-of-plane angular dependence of Δ*H*_*pp*_ can be well fitted according to Eq. (8) and Eq. (9) listed below, as shown by the solid line in Fig. 3(c), and the fitted value of *α* is 0.010. Detailed expressions for all the terms in Eq. (9) and the corresponding derivation process can be referred to Supplementary Material A.


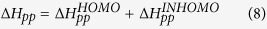



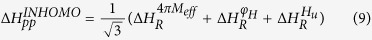


**Figure 4:**
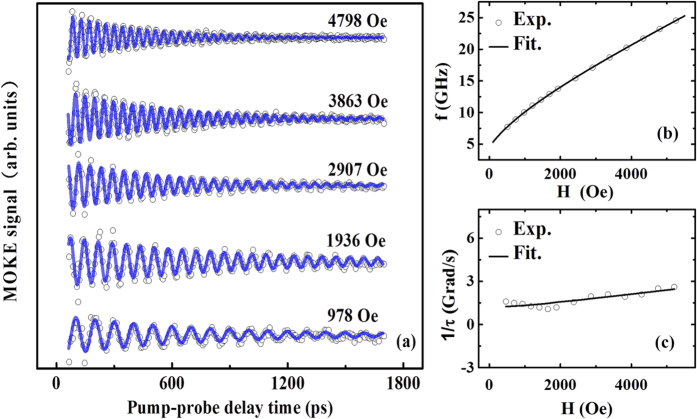
(**a**) The TRMOKE precession signals obtained under different applied magnetic fields, the dependences of (**b**) *f* and (**c**) 1/*τ* on the applied magnetic field for the CoFeB/GaAs(001) film. The open dots and solid lines represent the experimental and the fitted results, respectively.

Figure 4(a) shows the TRMOKE precession curves under various applied magnetic fields for the CoFeB/GaAs(001) film. Note that the TRMOKE precession signals are recorded at *φ*_H_ = 30°. Generally, the experimental TRMOKE curve can be analyzed by the following formula^18^


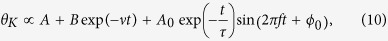


where A, B and ν are the offset, the background magnitude and the background recovery rate, respectively; A_0_, *f*, τ and *ϕ*_0_ denote precession amplitude, precession frequency, reversal lifetime and initial phase, respectively. The second term in Eq. (10) represents the decaying background of the magnetization, which has been abandoned in the present experimental results. As indicated by the blue lines shown in Fig. 4(a), the experimental TRMOKE results can be well fitted according to Eq. (10) with removing the second (decaying) term, and the values of *f* and 1/τ under different applied field (*H*) can be obtained consequently. The fitted values of *f* and 1/*τ* as functions of *H* are displayed in Fig. 4(b) and (c), respectively. Also by using the least square method, the *H* dependence of *f* can be well fitted according to Eq. (3) and Eq. (5), as exhibited by the solid lines in Fig. 3(b), and thus the fitted parameter can be achieved as 4π*M*_eff_ = 8667 Oe, *H*_u_ = 253 Oe and *g* = 2.18. On the other hand, according to Eqs (3, 4, 5) and considering that the precession frequency dispersion is induced by the dispersion of *M*_*eff*_ and *H*_u_ as those generally considered in the Δ*H* fitting for the FMR results, *τ* can be expressed as^17^,^18^,^25^


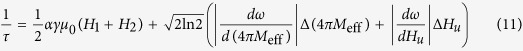


According to Eq. (11), the results of 1/*τ*~*H* can be fitted after inputting the values of 4π*M*_eff_, *H*_u_ and *g* obtained from the fitting of *f*~*H*, which can be seen by the solid line in Fig. 4(c). Therefore, the value of *α* can be obtained as 0.013.

## Discussion

It can be seen clearly that the value of the same parameter (e.g. *H*_u_, *g* or *α*) listed in Table 1 is different if different measuring method is adopted. Firstly, because the *g*-factor is a tensor, its value extracted by FMR in the case of in-plane configuration is slightly smaller than that of out-of-plane configuration in ultrathin films^26^. Secondly, in our latest work^27^, the value of *α* for the CoFeB(10 nm)/MgO film has been found to increase from 0.006 to 0.008 when the pump fluence is increased from 5 mJ/cm^2^ to 8 mJ/cm^2^, which is ascribed to the transient heating effect induced by the pump laser excitation. In the present CoFeB film, the *α* value obtained from TRMOKE is measured at a relatively high pump fluence of 8 mJ/cm^2^. Therefore, the transient heating effect may contribute to the enhancement of Gilbert damping and lead to the acquired *α* value (0.013) 30% larger than that (0.010) obtained by FMR. This effect may also cause a transient demagnetization of the CoFeB film and/or modify the interface between CoFeB and GaAs, which may result in the fitted value of *H*_u_ from TRMOKE (253 Oe) smaller than that from FMR (292 Oe). The fact that the *g* value acquired from TRMOKE (2.18) is larger than that acquired from FMR (2.13) may be also caused by the transient heating effect. To our knowledge, the *g* value is temperature dependent^28^,^29^. For example, the *g* value of Permalloy was found to be increased from ~2.07 to ~2.14 when the temperature was increased from liquid nitrogen temperature to room temperature^28^. Therefore, since the transient heating effect occurs in the TRMOKE measurement as mentioned above^27^, the sample may get a transient temperature rising, leading to the *g* value acquired by TRMOKE a little bit larger than that obtained by FMR. In addition, as shown in Table 1, no matter the *g* value is acquired from FMR or TRMOKE, it is obviously larger than that for the in-plane magnetized CoFeB films (*g*~2.07)^12^,^30^. Since the ratio between the orbit and spin magnetic moment can be described as *μ*_*L*_/*μ*_*S*_ = (*g* − 2)/2^26^,^28^, larger *g* value means larger orbit magnetic moment, which is well consistent with our newly obtained XMCD results^31^. The reason why the orbit magnetic moment is significantly enhanced may be resulted from the interfacial interaction between CoFeB and GaAs, which is similar to ultrathin Fe film epitaxially deposited on GaAs (100) substrate^32^.

To our knowledge, the *α* value for the CoFeB film is strongly dependent on the film thickness^13^,^30^. For example, an empirical *α*~1/*t* relationship is satisfied in the CoFeB/MgO film^13^. However, even for the same thickness of the CoFeB film, the *α* value in the present Ta/CoFeB(3.5 nm)/GaAs(001) film (0.010 for FMR and 0.013 for TRMOKE) is also significantly larger than that in the Co_40_Fe_40_B_20_/MgO film (~0.0053)^13^. It is well known that the intrinsic Gilbert damping is closely related to the spin-orbit coupling strength *ξ*^33^,^34^,^35^,^36^, which also determines the magnetic anisotropy. According to the theoretical Kamberský torque-correlation model^33^, *α* is predicted to be proportional to *ξ*^2^ (*ξ*^3^) at high (low) temperature region, which is mainly caused by the interband (intraband) transition. Very recently, P. He *et al*.^34^ have proved experimentally that *α* is proportional to *ξ*^2^ at room temperature by carrying out the Gilbert damping studies in FePdPt ternary alloys with fixed Fe content and varying the relative composition ratio between Pd and Pt. As listed in Table 1, the PMA field (*H*_P_ = 4π*M*_s_ −4π*M*_eff_) and the in-plane UMA field (*H*_u_) are about 6000 Oe and 300 Oe, respectively, which are both significantly larger than those cases if the CoFeB film is directly deposited on Si, MgO or other substrates^15^,^27^. Therefore, the increase of *α* in the present CoFeB film is mainly resulted from the strong spin-orbit coupling generated from the CoFeB/GaAs(001) interface, although the spin pumping effect originated from the CoFeB/Ta interface may also enhance the Gilbert damping a little bit^6^,^7^. On the other hand, in order to reveal the relationship between the in-pane UMA and the intrinsic Gilbert damping, the GaAs(110)/CoFeB(3.5 nm)/Ta(2 nm) film was fabricated, which shows a much weaker *H*_u_ (~30 Oe) and a comparable *H*_P_ (~5800 Oe)^22^. By adopting the same FMR technique, the *α* value is obtained to be 0.010, which is exactly the same as that in the same thick CoFeB film deposited on the GaAs (001) substrate. As mentioned above, since *H*_P_ (~6000 Oe) is about twenty times larger than *H*_u_ (~300 Oe), the spin-orbit coupling should play dominant role in determining the PMA. Therefore, although the in-plane UMA is much stronger than those for ordinary CoFeB films, it is weakly correlated to the spin-orbit coupling and thus has negligible contribution to the enhancement of *α*. This result unambiguously shows that although the in-plane UMA can be significantly enhanced if CoFeB film is deposited onto GaAs (001) substrate, the *α* value can be well kept.

In summary, we have studied the magnetization dynamics of ultrathin CoFeB film deposited on GaAs (001) substrate with significant in-plane UMA through FMR and TRMOKE techniques assisted by the magnetostatic measurements. All the FMR spectra and TRMOKE precession signals can be well fitted, and the reliable values of the parameters such as 4π*M*_eff_, *H*_u_, *g* and *α* can be acquired accordingly. The slight differences between the FMR and TRMOKE fitted results are ascribed to the transient heating effect during the TRMOKE measurements. In comparison with ordinary CoFeB films with negligible magnetic anisotropies, the intrinsic Gilbert damping constant is obviously increased in the CoFeB/GaAs(001) film, indicating strong interfacial spin-orbit coupling. Although the significant in-plane UMA is also resulted from the interfacial spin-orbit coupling, it plays minor role in the enhancement of *α* because its strength is much less than that of PMA. This result may be helpful for designing high-performance spintronic devices with strong in-plane UMA and comparatively low Gilbert damping.

## Methods

The commercial GaAs(001) wafers were used with the major-flat direction along [110] and the secondary-flat direction along [1–10]. These wafers were diced into 4 mm × 4 mm pieces as substrates. Before deposition of the CoFeB films, the surface of each substrate needs to be etched and cleaned by proper procedures, which can be referred to our previous report^22^. At room temperature, the CoFeB films were deposited on GaAs (001) substrates by dc magnetron sputtering at normal incidence from a commercial Co_56_Fe_24_B_20_ alloy target. A Ta film of 2 nm was deposited as capping layer to prevent the CoFeB film from oxidation. The base pressure was lower than 8.0 × 10^−6^ Pa and the Ar pressure kept at 0.3Pa during film deposition. The X-ray diffraction (XRD) patterns do not show any obvious peaks of Fe, Co or FeCo alloys in all the CoFeB films with thickness varied from 3.5 nm to 20 nm, indicating amorphous structures. The in-plane and out-of-plane magnetic hysteresis (*M*-*H*) loops were measured by a SQUID-VSM (Quantum Design) and a vector vibrating magnetometer (VVSM, Microsense EV7), respectively. The in-plane and out-of-plane FMR spectra in the form of derivative absorption are recorded at 9.78 GHz. The TRMOKE was performed to study the laser-excited magnetization precession using a pulsed Ti: sapphire regenerative amplifier. The pump laser wavelength, spot diameter, and energy density are 800 nm, 500 um and 8 mJ/cm^2^, respectively. And the probe laser wavelength, spot diameter, and pulse width are 400 nm, 100 um and 50 fs, respectively. It should be emphasized that all the measurements in this report were performed at room temperature.

## Additional Information

**How to cite this article:** Tu, H. Q. *et al*. Gilbert damping in CoFeB/GaAs(001) film with enhanced in-plane uniaxial magnetic anisotropy. *Sci. Rep.* **7**, 43971; doi: 10.1038/srep43971 (2017).

**Publisher's note:** Springer Nature remains neutral with regard to jurisdictional claims in published maps and institutional affiliations.

## Supplementary Material

Supplementary Material

## Figures and Tables

**Table 1:** All the fitted parameters achieved from different measurements for the CoFeB/GaAs(001) film.

Measurements	4π*M*_eff_ (Oe)	*H*_u_ (Oe)	*g*	*α*
In-plane ***H***_r_~*θ*_H_ (FMR)	8411	313	2.11	
Out-of-plane***H***_r_~*φ*_H_ (FMR)	8362	292	2.13	
Out-of-plane Δ***H***~*φ*_H_ (FMR)	/	/	/	0.010
*f*~***H*** (TRMOKE)	8667	253	2.18	
1/τ~***H*** (TRMOKE)	/	/	/	0.013
