# Supplementary material for: Gilbert damping in CoFeB/GaAs(001) film with enhanced in-plane uniaxial magnetic anisotropy
Source: Sci Rep. 2017 Mar 6;7:43971. doi: 10.1038/srep43971 (PMC5338288; doi:10.1038/srep43971)
Supplement: Supplementary Material [file srep43971-s1.pdf]

Gilbert damping in CoFeB/GaAs(001) film with enhanced in-plane uniaxial magnetic anisotropy

H. Q. Tu[1, 2], B. Liu[3], D. W. Huang[3], X. Z. Ruan[3], B. You[1,5,*], Z. C. Huang[4], Y. Zhai[4], Y. Gao[1], J. Wang[1], L. J. Wei[1], Y. Yuan[1], Y. B. Xu[3], J. Du[1,5,*]

[1]*National Laboratory of Solid State Microstructures and Department of Physics, Nanjing University, Nanjing 210093, P. R. China*

[2]*Department of Mathematics and Physics, Nanjing Institute of Technology, Nanjing 211167, P. R. China*

[3]*School of Electronic Science and Engineering, Nanjing University, Nanjing 210046, P. R. China*

[4]*Department of Physics and Jiangsu Key Laboratory of Advanced Metallic Materials, Southeast University, Nanjing 211189, P. R. China*

[5]*Collaborative Innovation Center of Advanced Microstructures, Nanjing 210093, P. R. China*

---

*Authors to whom correspondence should be addressed. Electronic addresses: youbiao@nju.edu.cn and jdu@nju.edu.cn

SUPPLEMENTARY MATERIAL

A. Derivation of the out-of-plane angular dependence of FMR spectrum linewidth ΔH_{pp}

After solving the LLG equation, one can achieve the intrinsic part of the linewidth, ΔH_{pp}^{HOMO}, for the angular dependent out-of-plane FMR spectra as described by the following equation [S1, S2]

$$\Delta H_{pp}^{HOMO} = \frac{\gamma\alpha\left[F_{\theta\theta} + F_{\varphi\varphi}(\sin^2\theta)^{-1}\right]}{\sqrt{3}M_S\left(\frac{d\omega}{dH}\right)} \qquad (1)$$

Here, θ and φ represent θ_M and φ_M respectively, which are illustrated in Fig. 1(a) in the manuscript; F denotes the total energy per unit volume and its expression can be found in Eq. (2) in the manuscript, and $F_{\theta\theta}= \partial^2F/\partial\theta^2$, $F_{\varphi\varphi} = \partial^2F/\partial\varphi^2$. Moreover, for the study of the out-of-plane angular dependence of FMR linewidth, the external magnetic field H is applied in the yz-plane, thus $\theta_H = \theta_M = \pi/2$. The expression of $\frac{d\omega}{dH}$ can be obtained by the first order Taylor expansion in combination with Eq. (3) and Eq. (5) in the manuscript, and finally ΔH_{pp}^{HOMO} can be written as

$$\Delta H_{pp}^{HOMO} = \frac{2\omega G}{\sqrt{3}\gamma^2 M_S}\frac{H_1+H_2}{\left(H_1+H_2\right)\cos\left(\varphi_H-\varphi_M\right)+\left(H_{2\varphi}+\frac{H_2H_{1\varphi}}{H_1}\right)\sin\left(\varphi_H-\varphi_M\right)}, \qquad (2)$$

where $G=\alpha\gamma M_S$, $H_1 = H\cos\left(\varphi_H-\varphi_M\right)+(4\pi M_{\mathrm{eff}}+H_u)\cos\left(2\varphi_M\right)$,

$H_{1\varphi} = H\sin\left(\varphi_H-\varphi_M\right)-2(4\pi M_{eff}+H_u)\sin\left(2\varphi_M\right)$,

$H_2 = H\cos\left(\varphi_H-\varphi_M\right)-4\pi M_{eff}\sin^2\varphi_M+H_u\cos^2\varphi_M$,

$H_{2\varphi} = H\sin\left(\varphi_H-\varphi_M\right)-\left(4\pi M_{eff}+H_u\right)\sin\left(2\varphi_M\right)$, $4\pi M_{eff}=4\pi M_S-H_P$, $\gamma=g\mu_B/\hbar$.

The extrinsic contribution to ΔH, termed as ΔH^{INHOMO}, is considered as the

fluctuations of $4\pi M_{\text{eff}}$, φ_{H} and H_{u}, which are resulted from structural inhomogeneity and defects present in thin magnetic films [S2, S3]. Similarly, by the first order Taylor expansion in combination with Eq. (3) and Eq. (5) in the manuscript, one can obtain the following expressions as

$$\Delta H_R^{4\pi M_{eff}} = \frac{H_1 \sin^2(\varphi_M) - H_2 \cos^2(\varphi_M) + \frac{1}{2}\left(H_{2\varphi} + \frac{H_2 H_{1\varphi}}{H_1}\right)\sin(2\varphi_M)}{(H_1 + H_2)\cos(\varphi_H - \varphi_M) + \left(H_{2\varphi} + \frac{H_2 H_{1\varphi}}{H_1}\right)\sin(\varphi_H - \varphi_M)} \Delta(4\pi M_{eff}) \quad (3)$$

$$\Delta H_R^{\varphi_H} = \frac{H(H_1 + H_2)\sin(\varphi_H - \varphi_M) - H\left(H_{2\varphi} + \frac{H_2 H_{1\varphi}}{H_1}\right)\cos(\varphi_H - \varphi_M)}{(H_1 + H_2)\cos(\varphi_H - \varphi_M) + \left(H_{2\varphi} + \frac{H_2 H_{1\varphi}}{H_1}\right)\sin(\varphi_H - \varphi_M)} \Delta\varphi_H \quad (4)$$

$$\Delta H_R^{H_u} = \frac{\frac{1}{2}\left(H_{2\varphi} + \frac{H_2 H_{1\varphi}}{H_1}\right)\sin(2\varphi_M) - \left(H_1 \cos^2(\varphi_M) + H_2 \cos(2\varphi_M)\right)}{(H_1 + H_2)\cos(\varphi_H - \varphi_M) + \left(H_{2\varphi} + \frac{H_2 H_{1\varphi}}{H_1}\right)\sin(\varphi_H - \varphi_M)} \Delta H_u \quad (5)$$

Therefore, the overall FMR spectrum linewidth ΔH_{pp} is the summation of ΔH_{pp}^{HOMO}, $\Delta H_R^{4\pi M_{eff}}$, $\Delta H_R^{\varphi_H}$ and $\Delta H_R^{H_u}$, which can be described by Eq. (8) and Eq. (9) in the manuscript. By fitting the experimental angular dependent data of ΔH_{pp} using these two equations, the intrinsic damping constant α and the values of $\Delta(4\pi M_{\text{eff}})$, $\Delta\varphi_{\text{H}}$ and ΔH_{u} as well can be obtained consequently.

References

[S1] Suhl, H. Ferromagnetic resonance in nickel ferrite between one and two kilomegacycles. *Phys. Rev.* **97**, 555 (1955).

[S2] Dubowik, J., Załeski, K., & Głowinski, H. Angular dependence of ferromagnetic resonance linewidth in thin films. *Phys. Rev. B* **84**, 184438 (2011).

[S3] Farle, M., Ferromagnetic resonance of ultrathin metallic layers. *Rep. Prog. Phys.* **61**, 769 (1998).
